# Robust neuronal differentiation of human embryonic stem cells for neurotoxicology

**DOI:** 10.1016/j.xpro.2022.101533

**Published:** 2022-09-16

**Authors:** Athina Samara, Martin Falck, Mari Spildrejorde, Magnus Leithaug, Ganesh Acharya, Robert Lyle, Ragnhild Eskeland

**Affiliations:** 1 Division of Clinical Paediatrics, Department of Women’s and Children’s Health, Karolinska Institutet, 17177 Stockholm, Sweden; 2 Astrid Lindgren Children′s Hospital Karolinska University Hospital, 17177 Stockholm, Sweden; 3 Department of Biosciences, University of Oslo, Blindern, PO Box 1066, 0316 Oslo, Norway; 4 PharmaTox Strategic Research Initiative, Faculty of Mathematics and Natural Sciences, University of Oslo, 0316 Oslo, Norway; 5 Department of Medical Genetics and Norwegian Sequencing Centre, Oslo University Hospital, Kirkeveien 166, 0450 Oslo, Norway; 6 Institute of Clinical Medicine, Faculty of Medicine, University of Oslo, 0450 Oslo, Norway; 7 Division of Obstetrics and Gynecology, Department of Clinical Science, Intervention and Technology (CLINTEC), Karolinska Institutet, Alfred Nobels Allé 8, 14152 Stockholm, Sweden; 8 Center for Fetal Medicine, Karolinska University Hospital Huddinge, 14186 Stockholm, Sweden; 9 Centre for Fertility and Health, Norwegian Institute of Public Health, PO 222 Skøyen, 0213 Oslo, Norway; 10 Institute of Basic Medical Sciences, Department of Molecular Medicine, Faculty of Medicine, University of Oslo, Blindern, PO Box 1112, 0317 Oslo, Norway

**Keywords:** Cell culture, Developmental biology, Gene Expression, Neuroscience, Stem Cells, Cell Differentiation

## Abstract

Here, we describe a protocol for rapid neuronal differentiation from human embryonic stem cells (hESCs) toward a heterogenous population of telencephalic progenitors, immature and mature neurons, for drug-screening and early-brain differentiation studies. hESC neuronal differentiation depends on adhesion and minimal cell-passaging to avert monolayer cross-connectivity rupture. In this protocol, we detail optimized cell-seeding densities and coating conditions with high cell viability suitable for neurotoxicology and high-resolution single-cell omics studies. Daily media changes reduce compound instability and degradation for optimal screening.

For complete details on the use and execution of this protocol, please refer to Samara et al. (2022).

## Highlights

• Day-by-day visual aids to monitor morphological changes during differentiation
• Optimized cell numbers and passages for neurotoxicology studies
• Web tools available for single-cell omics data
• Ideal for early brain development exploration from human embryonic stem cells

## Abstract (editor highlights)

Publisher’s note: Undertaking any experimental protocol requires adherence to local institutional guidelines for laboratory safety and ethics.

## Graphical abstract

**Figure:**
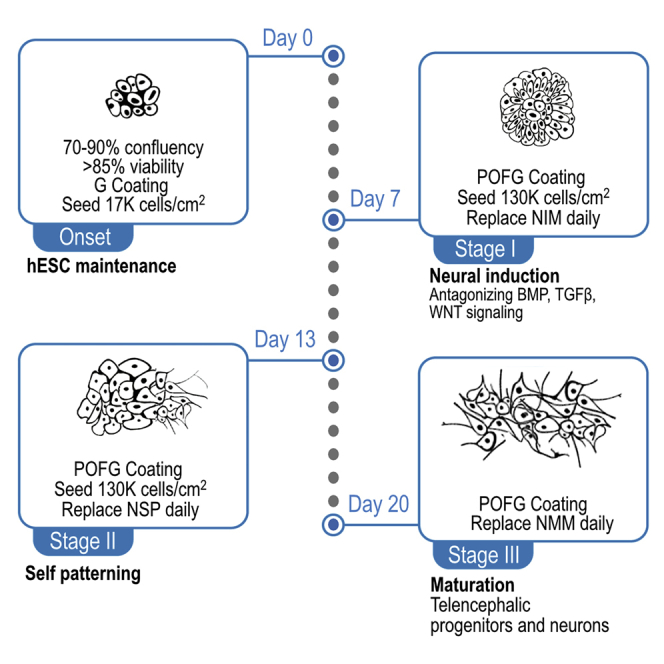

## Before you begin

The neuronal differentiation protocol below describes the specific steps for using the hESC cell line HS360 (Ström et al., 2010; Main et al., 2020), and this has been replicated using H9 hESCs (Thomson et al., 1998) under the same conditions. hESCs should be maintained under pluripotency conditions before neural differentiation, and protocols for hESC maintenance are standardized (Desai et al., 2015). The protocol consists of three major stages: neural induction, self-patterning and neuronal maturation. Cell counts are standardized at Day 0 seeding and the culture medium is changed daily. The cell cultures are split and reseeded at standardized cell numbers in differently coated culture dishes at Day 7 and Day 13, and the protocol ends at Day 20. The preferred cell culture format is 12-well culture dishes.

We provide detailed recipes for the preparation of the stock and working solutions, and for the composition of the media used at all stages in materials and equipment. The recommended volumes for the coating of the culture plates, cell detachment and washing, along with cell culture media volumes and cell seeding counts are provided in Table 1. The identifiers and source of the reagents used throughout the protocol can be found at the key resources table.

**Table 1:** Recommended cell culture media, coating, cell detachment and washing volumes, and cell seeding counts

	12 - well format	24 - well format
Base matrix coating volume	0.5 mL/well	0.3 mL/well
Cell culture medium volume	1 mL/well	0.5 mL/well
Volume of 1× PBS used for washing	1 mL/well	0.5 mL/well
Volume of Accutase used for cell detachment	1 mL/well	0.5 mL/well
Day 0 cell seed counts	17K cells/cm^2^	
Day 7 cell seed counts	130K cells/cm^2^	
Day 13 cell seed counts	130K cells/cm^2^	

The same conditions apply and have been tested with glass coverslips.

### Preparation at onset of protocol: Cells and reagents

**Timing: 1.5 h, including coating of culture dishes**

1. Ensure that you have enough hESC cells in culture, growing at a maximum confluency of 80%–90% before seeding.
2. Prepare Stage I neural induction medium (NIM). See manufacturer’s instructions for N2 supplement: if N2 is aliquoted, and the penicillin/streptomycin solution thawed, the required time is 10 min.
3. Precoat culture dishes with Geltrex for stage I of the protocol. See also recipes for preparation of stock solutions.

## Key resources table

**Table:** 

REAGENT or RESOURCE	SOURCE	IDENTIFIER
**Antibodies**		

FOXG1 (1:1000)	Sigma-Aldrich	SAB2102981
AF488 Goat anti-Rabbit (1:1500)	Thermo Fisher Scientific	A32731
Nestin AF488-conjugated, 10C2 (1:500)	Santa Cruz Biotech	SC23927
PAX6 AF647-conjugated (1:500)	Santa Cruz Biotech	SC81649
SOX2 AF647-conjugated, E-4 (1:250)	Santa Cruz Biotech	SC365823
TUBB3 PE- conjugated, 2G10 (1:500)	Santa Cruz Biotech	SC80005

**Chemicals, peptides, and recombinant proteins**		

Geltrex™ LDEV-Free, hESC-Qualified, Reduced Growth Factor Basement Membrane Matrix	Thermo Fisher Scientific	A1413302
KnockOut™ DMEM	Thermo Fisher Scientific	10829018
PBS, no calcium, no magnesium	Thermo Fisher Scientific/GIBCO	14190
Accutase™ Cell Detachment Solution	STEMCELL Technologies	7920
UltraPure 0.5 M EDTA, pH 8.0	Thermo Fisher Scientific	15575020
RHO/ROCK Pathway Inhibitor Y-27632	STEMCELL Technologies	SCM075
Essential 8™ Medium	Thermo Fisher Scientific	A1517001
Poly-L-ornithine hydrobromide	Sigma-Aldrich/ Merck	P3655
Fibronectin (Bovine Protein, Plasma)	Thermo Fisher Scientific	33010018
N2 supplement (100×)	Thermo Fisher Scientific	17502048
Advanced DMEM/F-12	Thermo Fisher Scientific	12634028
GlutaMAX™ Supplement	GIBCO/ Thermo Fisher Scientific	35050061
Penicillin Streptomycin (10000 U/mL)	Thermo Fisher Scientific	15140122
**L**DN-193189	STEMCELL Technologies	72148
**S**B 431542 (hydrate)	Sigma-Aldrich/Merck	S4317
**X**AV939	STEMCELL Technologies	72674
B-27™ Supplement (50×), serum free	Thermo Fisher Scientific	17504044
Recombinant Human FGF basic	PeproTech	100-18B
Recombinant Human EGF, Animal-Free	PeproTech	AF-100-15
Invitrogen™ ProLong™ Gold Antifade Mountant with DAPI	Fisher Scientific/ Invitrogen	P36931
Paraformaldehyde	Sigma-Aldrich	158127
Triton X-100	Thermo Fisher Scientific	11332481001
Normal-Horse-Serum-Blocking-Solution	Bionordica/ Vectorlabs	S-2000-20
DMSO	Sigma-Aldrich	D2650-100ML

**Critical commercial assays**		

Countess™ Cell Counting Chamber Slides	Thermo Fisher Scientific	C10312
RNeasy Plus Mini - RNA extraction Kit	QIAGEN	74034
RNAse-Free DNase Set	QIAGEN	79254
RNA/DNA purification kit	Nordic biosite	298-48700
RNase-Free DNase I Kit	Nordic biosite	298-25720
Qubit™ RNA BR Assay Kit	Thermo Fisher Scientific/Invitrogen	Q10211
QuantiTect Reverse Transcription Kit	QIAGEN	205311
Taqman® Gene Expression Master Mix	Thermo Fisher Scientific	4369016

**Experimental models: Cell lines**		

Human embryonic cells, HS360 (passage numbers 40–60)	Stockholms Medicinska Biobank/Sweden	HS360
Human embryonic cells, H9 (passage numbers 40–60)	WiCell/ USA	WA09 (H9)

**Oligonucleotides**		

*POU5F1*	Thermo Fisher Scientific/TaqMan™	Hs00999632_g1
*SOX2*	Thermo Fisher Scientific/TaqMan™	Hs01053049_s1
*NANOG*	Thermo Fisher Scientific/TaqMan™	Hs04399610_g1
*NES*	Thermo Fisher Scientific/TaqMan™	Hs04187831_g1
*FOXG1*	Thermo Fisher Scientific/TaqMan™	Hs01850784_s1
*TUBB3*	Thermo Fisher Scientific/TaqMan™	Hs00801390_s1
*MAP2*	Thermo Fisher Scientific/TaqMan™	Hs00258900_m1
*PAX6*	Thermo Fisher Scientific/TaqMan™	Hs00240871_m1
*OTX2*	Thermo Fisher Scientific/TaqMan™	Hs00222238_m1
*VIM*	Thermo Fisher Scientific/TaqMan™	Hs00958111_m1
*NEUROD1*	Thermo Fisher Scientific/TaqMan™	Hs01922995_s1
*RPL30*	Thermo Fisher Scientific/TaqMan™	Hs00265497_m1
*RAF1*	Sigma-Aldrich	Universal Probe Library/ Probe 56 4688538001

**Other**		

Microscope: EVOS® FL Cell Imaging System	Thermo Fisher Scientific	AMF4300
Cell counter: Countess® II FL Automated Cell Counter	Thermo Fisher Scientific	MQAF1000
Nucleic acid Quantification: Qubit Fluorometer	Thermo Fisher Scientific/Life Technologies	2.0
Nucleic Acid Quality analysis system: Bioanalyzer	Agilent	2100
For Gene expression analysis: QuantStudio Real-Time PCR System	Thermo Fisher Scientific/Applied Biosystems	12K Flex

**Software and algorithms**		

ddCt Algorithm for the Analysis of Quantitative Real-Time PCR (qRT-PCR) ddCt package v.1.40.0 (visualized using the tidyverse package in R 4.0.4)	Zhang et al. (2021)	https://bioconductor.org/packages/3.14/bioc/html/ddCt.html https://rdrr.io/r/utils/install.packages.html
Statistical comparisons in R using t-test in ggpubr package v.0.4.0	Kassambara (2020)	https://rpkgs.datanovia.com/ggpubr/

## Materials and equipment

**Table:** Base medium

Reagent	Final concentration	Amount
Advanced DMEM/F12		485 mL
GlutaMAX Supplement	1%	5 mL
Penicillin / Streptomycin	1%	5 mL
N2 Supplement	1%	5 mL
**Total**		**500 mL**

Store at 4°C for up to two weeks.

**Table:** Stage I: NIM (neural induction medium)

Reagent	Final concentration	Amount for 1 12-well culture dish
Base medium		13 mL
SB431542, 20 mM (added daily)	10 μM	6.5 μL
LDN-193189, 1 mM (added daily)	100 nM	1.3 μL
XAV939, 20 mM (added daily)	2 μM	1.3 μL
**Total**		**13 mL**

Suggested use after addition of SB431542, LDN-193189, XAV939: same day.

**Table:** Stage II: NSPM (neuronal self-patterning medium)

Reagent	Final concentration	Amount
Base medium		49.5 mL
B27 Supplement	1%	0.5 mL
**Total**		**50 mL**

Store at 4°C for up to two weeks.

**Table:** Stage III: NMM (neuronal maturation medium)

Reagent	Final concentration	Amount
Base medium		49.75 mL
B27 Supplement	0.5%	0.25 mL
**Total**		**50 mL**
		**Amount for 1 12-well culture dish**
Base medium + B27		13 mL
FGF2, 10 μg/mL (added daily)	10 ng/mL	13 μL
EGF, 10 μg/mL (added daily)	10 ng/mL	13 μL
**Total**		**13 mL**

Store Base medium supplemented with B27 at 4°C for up to two weeks. Suggested use after addition of FGF2 and EGF: same day.

## Step-by-step method details

### Stage 0: hESC maintenance

**Timing: 3 days**

This section describes hESC culture and maintenance in E8 medium on Geltrex coated 6 well dishes. There are alternative culture methods for hESCs and induced pluripotent stem cells available. Refer to the preferred protocol of choice.

1. Coat a well of a 6 well dish with Geltrex solution (2 mL per well) and place in the incubator for 1 h.
2. Thaw a vial of hESCs, and transfer cell suspension from cryovial to 2 mL of prewarmed Essential 8 medium containing 10 μM ROCK inhibitor (E8/ROCKi).
3. The cells are pelleted (300 × *g* for 4 min), the supernatant is removed, and the pellet is resuspended again in 2 mL E8/ROCKi medium.
4. Remove Geltrex solution and plate cells using a 10 or 5 mL pipette.
5. Change culture medium (E8) daily.
6. Passage the cells 1:3 when they reach 70%–90% confluency.
  a. To passage cells, aspirate media, wash briefly with 2 mL of PBS (without Ca2+ and Mg2+) and remove the PBS wash.
  b. Add 1 mL of room temperature 0.5 mM EDTA solution (stored at room temperature) to the well and incubate at room temperature for 3–5 min, or until cells begin to uniformly detach.
  c. Collect cell suspension using a 10 or 5 mL pipette in a 15 mL tube, pellet cells at 300 × *g* for 4 min, remove supernatant and resuspend in 6 mL of E8 medium.
  d. Plate cells in 3 wells (6 well culture dish) precoated with Geltrex.
7. Incubate cells at 37°C/ 5% CO_2._

**CRITICAL:** Cell viability must be higher than 85% at passaging.

### Stage I: Neural induction

**Timing: 7 days**

**Figure 1:**
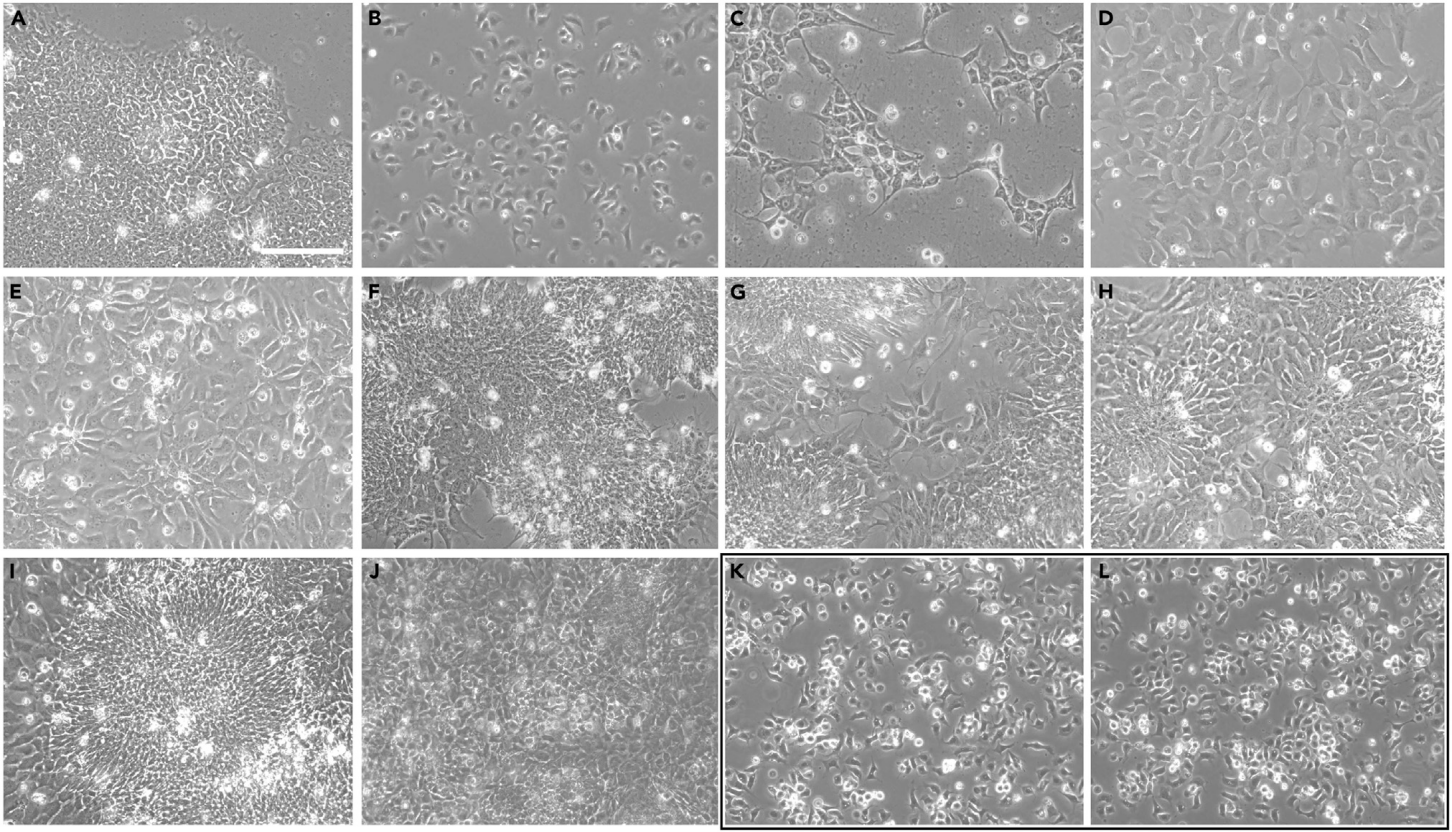
A series of representative brightfield images demonstrating the characteristic formation of rosettes during stage I neural induction (A) The protocol starts with the addition of Accutase to the hESC cells in culture (A), and cell counts and seeding for Stage I initiation. (A) Characteristic morphology of the hESCs with the small cytoplasm growing in E8 medium showing no signs of spontaneous differentiation. (B) Day 0, showing the cells 1 h after plating. (C) Representative image of the culture at Day 1, demonstrating the grid-like formation typical after ROCKi supplementation at Day 1. (D and E) Proliferation and confluency are enhanced at Day 2 and already at Day 3 the initiation of the columnar arrangement of rosettes can be observed (E). (F–H) The formation and maturation of rosettes and their compaction to neural tube-like structures is the characteristic feature of this part of the protocol. Although some less populated areas may be observed, (as shown at the Day 5 image, (G), other than few areas at the borders of the culture wells, the cells are highly confluent without viability issues (as assessed by the cell counts). (I) Rosettes at this stage may be looser (H) or reach high compaction (I). (J) At day 7 the cells are collected to be transitioned to Stage II. Images were taken with an EVOS FL microscope at 20× magnification and scale bar corresponds to 100 μm. **Pause point.** (K and L) Cells can be collected and frozen at this point (Day 7). The black-framed panel shows the cells 1 h after passaging (K), if the protocol continues uninterrupted or if cells are frozen and reseeded at a later stage (L). Images were taken with an EVOS FL microscope at 20× magnification and scale bar corresponds to 100 μm.

Day 0 is the protocol initiation day, and Day 1 is the neural induction initiation day. Times of media changes should be noted and reproduced as accurately as possible throughout the protocol. Radial patterning is visible in the cell monolayer from day 3, and by day 6 neural rosettes should be clearly visible (Figure 1). Day 7 marks the end of the neuronal induction part of the protocol and cells are passaged to the self-patterning Stage II.

#### Preparation of culture dishes and culture media

8. Refer to materials and equipment-section for reagents and NIM medium composition instructions.
9. Ensure that culture dishes are properly coated with Geltrex (Table 2). Add 0.5 mL Geltrex solution per well for 12-well culture dishes and place in the incubator for 1 h.

**Table 2:** Growth factors, cytokines, small molecules, and coating solution stock and working solution preparation

SB 431542 (hydrate) To achieve a final <0.1% DMSO concentration in the culture medium, dissolve SB431542 in DMSO at highest recommended concentration. • Dissolve 5 mg SB431542 in 650 μL DMSO for a stock concentration of 20 mM. • Stock to be aliquoted according to experimental design. For example, if 20 mL of culture media are to be used daily, 10 μL aliquots are prepared and stored at −20°C. • Use a final concentration of 10 μM in culture medium (1:2,000, 0.5 μL/mL media).	LDN-193189 • Dissolve 10 mg LDN-193189 in 22.6 mL DMSO for a stock concentration of 1 mM. • Place tube(s) in water bath (37°C), with occasional vortexing until the compound dissolves, making sure there are no undissolved particles. • Stock to be aliquoted in volumes suiting the experimental design (for example, if 20 mL of culture media are to be used daily, 2 μL aliquots are prepared and stored at −20°C. • Use a final concentration of 100 nM in culture medium (1:10,000); dilute once more 1:10 in ddH_2_O for a more convenient 1:1,000 working stock solution.
XAV939 • Dissolve 10 mg XAV in 1.6 mL DMSO for a 20 mM stock concentration. • Aliquot stock and store at −20°C. • Use a final concentration of 2 μM in culture medium (1:10,000); dilute 1:10 in ddH_2_O for a more convenient 1:1,000 work stock.	RHO/ROCK Pathway inhibitor Y-27632 • Dilute to 10 mM in ddH_2_O • Small aliquots may be stored at −20°C, avoid repeated freeze-thaw cycles. • Use a final concentration of 10 μM in culture medium (1:1,000)
Recombinant human FGF basic Centrifuge vial containing powder prior to opening. Once dissolved, store at 4°C for 1 week, or −20°C to −80°C for long-term storage. Avoid freeze/thaw cycles; recommended storage time in this form and temperature is approximately 12 months. • Reconstitute in 1× PBS 0.1% BSA to a bFGF concentration of 10 μg/mL. Do not vortex. • Aliquot stock solution: e.g., 25 μL into 25 mL of culture medium (if used at 1:1,000). • Use a final concentration of 10 ng/mL in culture medium.	Recombinant human EGF basic • Once dissolved, store at 4°C for 1 week, or −20°C to −80°C for long-term storage. Dissolve 1,000 μg in 10 mL of 1× PBS (0.1% BSA added for long-term storage). This results in a 100 μg/mL solution (10,000× stock) • Aliquot 10 mL volume into suitable smaller volumes, for example 100 μL, and store at −20°C. • Use a master/stock aliquot to make a 10× dilution and prepare working solution aliquots (for example, use 100 μL and add 900 μL of 1× PBS (0.1% BSA) to make a 10× 100 μL aliquot 1:1000 working solution. • Use final concentration of 10 ng/mL in culture medium.
Poly-L-ornithine hydrobromide Prepare in cell culture grade water by dissolving powder to a 10 mg/mL solution, dilute to 0.1 mg/mL with ddH_2_O, and further dilute 1:500 for the stock solution. The stock solution can be stored at 4°C for several weeks, and at −20°C for long-term storage.	Geltrex basement membrane matrix • Thaw stock vial in a beaker containing ice, in a 4°C refrigerator overnight (12–16 h). • Keep tubes on ice while making aliquots. • Ready to use Geltrex working solution is prepared by adding the 250 μL stock in a 50 mL Eppendorf tube containing 24.75 mL of Knockout DMEM. Aliquot 250 μL of working solution in 0.5 mL tubes. • For coating, a stock solution aliquot is thawed on ice or at 4°C and diluted 1:100 in serum-free media (see above). • Coat using a volume that will cover the surface of the well/plate (e.g., 1 mL for a well in a 6-well plate); tilt culture plate sideways till surface is covered, making sure there are no dry patches. • Place culture plate in incubator (37°C) for 1 h, before use. • Remove Geltrex solution and seed cell suspension. Precoating: for long-term storage of coated plates, seal plates with parafilm and store at 4°C for a maximum of 2 weeks. Before use, check that surface is covered and that Geltrex has not solidified or dried out.
Fibronectin Dissolve contents of vial in cell culture grade water. Dilute to 1 mg/mL in ddH_2_O by gently warming the vial to 37°C. This is further used as a 1:1,000 stock solution. Do not agitate as it may cause precipitation. Store stock solution at −20°C.	
N2 and B-27 thawing and aliquoting See manufacturer’s instructions for N2 and B-27 supplements.

#### Step-by-step stage I instructions

Day 0.

10. Starting with 80%–90% confluent, undifferentiated hESCs, aspirate medium, wash cells twice with 1× PBS to remove medium.
11. Aspirate PBS wash each time, before adding 1 mL prewarmed Accutase per well. The optimal Accutase incubation time needed, to collect a cell suspension consisting of clumps of 1–10 cells for hESC lines HS360 and H9 and without vigorous pipetting, was empirically evaluated to be 7 min.
12. Place culture dish containing Accutase in the incubator at 37°C for 7 min.

***Note:*** Protocol efficiency relies on the suspension having a high cell viability at seeding (ideally above 85%, as determined by the report of the Countess II). As hESCs are sensitive to vigorous pipetting, the incubation time in Accutase can be extended by 2 min (to a total of 9 min) instead of using mechanical force to attain an easy to collect single-cell suspension.

13. Collect the cell suspension using a 5 mL pipette, as it has a large diameter similar to the size of hESCs and is optimal for passaging and viability.
14. Transfer the single-cell suspension to a 15 mL tube containing 4 mL Essential 8 medium containing 10 μM ROCK inhibitor (E8/ROCKi) and set aside 20 μL of the suspension for cell counting.
15. Pellet cells (300 × *g* for 4 min), remove the supernatant and resuspend pellet in 10 mL E8/ROCKi medium. (See notes on use of ROCKi at troubleshooting section).
16. While the cells are being centrifuged, count the cells and assess the viability using trypan blue in an automated cell counter, such as the Countess II. The cell count can also be performed using a standard haemocytometer.
17. Remove Geltrex solution from prewarmed Geltrex-coated culture dishes.
18. Immediately plate cells at 17K cells/cm^2^ on Geltrex-coated culture dishes. The preferred format is 12-well culture dishes. Follow even cell spreading instructions (see troubleshooting section).
19. Incubate cells at 37°C/5% CO_2._

Day 1.

20. On Day 1, replace 1 mL E8/ROCKi cell culture medium with the neural induction medium (NIM) containing the LSX inhibitors to initiate the neuronal differentiation induction of hESCS and generate cells of anterior neuroectodermal fate.

***Note:*** For culture work form Day 1–6, change NIM medium supplemented with LSX inhibitors (materials and equipment-section) daily.

***Note:*** Add LSX inhibitors to the prewarmed (37°C) NIM fresh before replacing culture medium.

***Note:*** Day 7 is the last day of Stage I.

**Pause point:** Cells can be collected and frozen at Day 7.

#### Notes about choice of components for neural induction

Neuronal induction from human pluripotent stem cells can be directed to generate cells of anterior neuroectodermal fate using three small molecules LDN, SB and XAV, that antagonize the BMP, TGF-ß and WNT signaling pathways at standardized (100 nM LDN, 10 μM SB, 2 μM XAV) (Ohashi et al., 2018; Cakir et al., 2019) or similar concentrations of these small molecules (Tchieu et al., 2017; Major et al., 2017). The serum-free, chemically defined N2 supplement, is used from Day 1 and throughout the differentiation protocol. N2 is essential for cell commitment and differentiation, but also for survival and expression of post-mitotic neurons in culture. It favors the expression of neural-specific genes (such as neural specific filaments, for example TUBB3 and NFH) and inhibits the growth of non-neuronal cells (or undifferentiated residual ES cells) in culture.

#### Brief description of the outcomes of stage I

We include a series of brightfield images to demonstrate how the cells, starting from the confluent hESC cultures at Day 0, differentiate towards neuroectoderm. Representative images are shown in Figure 1. A full timeline of representative images (at 20× magnification) of all staged of the protocol are also available (Figure 6). Cells at Day 7 can be collected and frozen (in NIM containing 10% DMSO), to be thawed and reseeded at a later stage.

**CRITICAL:** Cell viability must be higher than 85% at passaging.

**CRITICAL:** At this stage, differentiating cells are sensitive to disturbance but rapidly reach confluence. Thus, at daily cell culture medium changes, care should be taken not to disrupt the monolayer. To remove media, the culture dish is lifted from the back at an angle that lets the medium well-up at the front, and the pipettor is placed vertically, to permit medium removal without contact and without disturbing the cells. 90% of the medium is removed, so that the cells do not dry out while replacing media. To replace media, the front edge of the culture dish sits on the hood floor, tilted forward, and medium is changed placing the pipette tip at an angle, by the side at the wall of each well, adding the culture medium dropwise and slowly, protecting the monolayer from mechanical disruption.

### Stage II: Neuronal self-patterning

**Timing: 6 days**

After the Stage I fate induction, this second stage of the differentiation protocol is a growth factor-free, self-patterning stage where the neural precursor monolayer is maintained in N2/B27 supplemented medium. The cells remain adherent while survival and maturation are enhanced. Given that the differentiating cells are split and replated twice in 20 days, and seeded at high confluency from day 7 onwards, B-27 supplement is added at Stage II to aid neuronal outgrowth and long-term neuronal survival.

#### Preparation of culture dishes and culture media

**Timing: 3.5 h**

Refer to materials and equipment-section for reagent, coating and Neuronal Self Patterning Medium (NSPM) medium composition instructions.

21. Coating Instructions for cell culture plates used in Stage II.
  a. Prepare the POF mix diluting a stock polyornithine aliquot 1:500 in ddH_2_O (for a final concentration of 20 μg/mL) and add to that a fibronectin stock aliquot (for a final concentration of 1 μg/mL). To make a 50 mL POF solution, add 50 μL of the fibronectin stock solution.
  b. Add 0.5 mL of the POF mix to each well of a 12 well dish and incubate the plate at 37°C for 2 h.
  c. Wash each well carefully with 1 mL of 1× PBS and aspirate PBS wash.
  d. Add 0.5 mL of Geltrex solution and incubate at 37°C for 1 h.
    ***Note:*** In our hands, the combination of Geltrex over the POF coating, gave the highest viability in order to eliminate passaging steps at this seeding density.
  e. Plates are ready to be used. Alternatively, they can be stored at 4°C for up to two weeks.

22. Prepare Stage II NSP medium. See manufacturer’s instructions for N2 and B27 supplements. If N2 and B27 are aliquoted, and the penicillin/streptomycin solution thawed, the required time is 10 min.

#### Step-by-step stage II instructions

Day 7.

23. Ensure that culture dishes are properly coated and prewarmed in the cell incubator.
24. Aspirate culture medium add 1 mL prewarmed Accutase to each well and place culture dish in the incubator for 7 min.

***Note:*** Protocol efficiency relies on high viability of the cell suspension at seeding (ideally above 90%). As proliferating cells at high confluency may be sensitive to vigorous pipetting, incubation time in Accutase can be extended by 2 min (to a total of 9 min) instead of using mechanical force to attain an easy to collect single-cell suspension.

25. Collect cells in 15 mL tubes using a pipettor with 10 mL, 5 mL or 1 mL serological pipettes. At this stage, cells can also be collected with a single channel pipette using a 1 mL tip.
26. Transfer single-cell suspension to 15 mL tubes containing NSP medium with 10 μM ROCKi and set aside 20 μL of the suspension for cell counting.
27. Pellet cells (300 × *g* for 4 min), remove supernatant and resuspend the pellet in NSP/ROCKi medium.
28. While cells are being centrifuged, count the cells and assess the viability using trypan blue in Countess II. The cell count can also be performed using a standard haemocytometer.
29. Remove Geltrex solution from prewarmed POF-Geltrex (POFG)-coated culture dishes.
30. Immediately plate cells at 130K cells/cm^2^ (we generally use 12-well dishes). Follow even cell-spreading instructions (see troubleshooting section).
31. Incubate cells at 37°C/5% CO_2._

***Note:*** For culture work from Day 8 to Day 12, change NSPM daily.

***Note:*** ROCKi is only used at passaging cells at Day 7.

#### Brief description of the rationale and outcomes of stage II

Sensitivity to pharmacological treatments could be dramatically different depending on cell type, cell confluency and free space availability, or cell to cell contact inhibition. Unlike, functional studies of individual neurons that require low density for synapse formation and dendritic spine morphology analyses, neuronal induction protocols should employ a medium- to- high-density seeding approach at the cell-culture- split level, followed by higher density at replating, to permit optimal cell survival and maturation. Of note, hESCs, neuronal precursors and neurons are contact dependent cells, but contact inhibition is also a known factor affecting cell signaling cascades and gene expression patterns (Ribatti, 2017; Gérard and Goldbeter, 2014; Carmona-Fontaine et al., 2008). This makes the newly formed cell connections in a monolayer of differentiating cells fragile and susceptible to guidance cues. Thus, cell density at seeding and passaging should be taken under consideration to avoid repetitive mechanical disruption of cell connections due to multiple passaging steps. This is important both for the accurate morphological characterization of the chosen time points of analysis and for the future pharmacological studies (Biffi et al., 2013; Ge et al., 2015).

**Figure 2:**
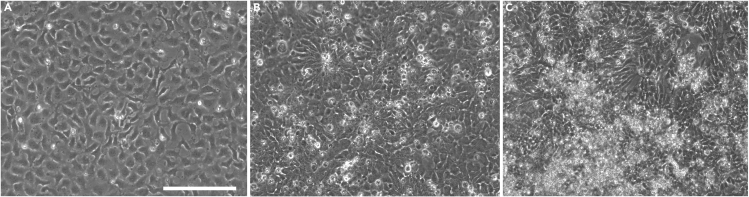
Representative phase contrast images at day 8, day 11 and day 13, showing how the neural stem cells and neuronal precursors (NSCs/NPCs) organize in the self-patterning stage (A) Image taken at day 8, the day after plating cells for Stage II. (B) The expanded cells, by day 11 have the morphology of neural progenitors. (C) By day 13 the culture forms as a heterogeneous cell population composed of precursors and immature neurons. All images were taken before routine media changes. As described, daily culture medium replacement is not accompanied with washing steps, thus by day 13, dead cells and debris may be accumulating. Images were taken with an EVOS FL microscope at 20× magnification and scale bar corresponds to 100 μm.

A set of representative phase contrast images at days 8, 11 and 13 showing how neural stem cells and neuronal precursors organize in the self-patterning stage are shown in Figure 2 (while the full Stage II image timeline from Day 7–13 is available at Figure 6).

**CRITICAL:** Cell viability must be higher than 85% at passaging.

### Stage III: Neuronal maturation

**Timing: 7 days**

In addition to the N2 supplement and the B27 supplement, the Stage III cell culture medium is supplemented with bFGF and EGF. B27 was empirically reduced by 50% compared to Stage II, as cells are preconditioned to the coating, and supplementation of culture medium with bFGF and EGF can promote growth of maturing neurons.

#### Preparation of culture dishes and culture media

**Timing: 3.5 h**

Refer to materials and equipment-section for reagent, coating and Neuronal Maturation Medium (NMM) composition instructions.

32. Coating Instructions for cell culture plates used in Stage III are the same as in Stage II.
  a. Prepare the POF mix diluting a stock polyornithine aliquot 1:500 in ddH_2_O (for a final concentration of 20 μg/mL) and add to that a fibronectin stock aliquot (for a final concentration of 1 μg/mL). To make a 50 mL POF solution, add 50 μL of the fibronectin stock solution.
  b. Add 5 mL of the POF mix to each well of a 12 well dish and incubate the plate at 37°C for 2 h.
  c. Wash each well carefully with 1 mL of 1× PBS and aspirate PBS wash.
  d. Add 0.5 mL of Geltrex solution and incubate at 37°C for another 1 h.
  e. Plates are ready to be used. Alternatively, they can be stored at 4°C for up to two weeks.
33. Prepare Stage III culture medium (NMM). If N2 and B27 are aliquoted and the penicillin/streptomycin solution is thawed, the time required is 10 min.

#### Step-by-step stage III instructions

Day 13.

34. Ensure that culture dishes are properly coated and prewarmed in the cell incubator.
35. Aspirate culture medium, add 1 mL prewarmed Accutase to each well and place culture dish in the incubator for 7 min at 37°C.

***Note:*** Protocol efficiency relies on high viability of the cell suspension at seeding (ideally above 90%). Incubation time in Accutase can be extended by 2 min (to a total of 9 min) instead of using mechanical force to attain an easy to collect single-cell suspension.

36. Collect cells using a pipettor with 10 mL, 5 mL or 1 mL serological pipettes. Here, cells can also be collected with a single channel pipettor using a 1 mL tip.
37. Transfer the single-cell suspension to 15 mL tubes containing NMM with 10 μM ROCKi and set aside 20 μL of the suspension for the cell count.
38. Pellet cells (300 × *g* for 4 min) remove supernatant and resuspend the pellet in NM/ROCKi medium.
39. While cells are being centrifuged, count the cells and assess the viability using trypan blue in Countess II. The cell count can also be performed using a standard haemocytometer.
40. Remove Geltrex solution from prewarmed POFG-coated culture dishes.
41. Immediately plate cells at 130K cells/cm^2^. Follow even cell-spreading instructions (see troubleshooting section).
42. Incubate cells at 37°C/5% CO_2._

***Note:*** For culture work from Day 14 to Day 19, change NMM daily.

***Note:*** ROCKi is only used at passaging cells at Day 13.

**CRITICAL:** Cell viability must be higher than 85% at passaging.

**Figure 3:**
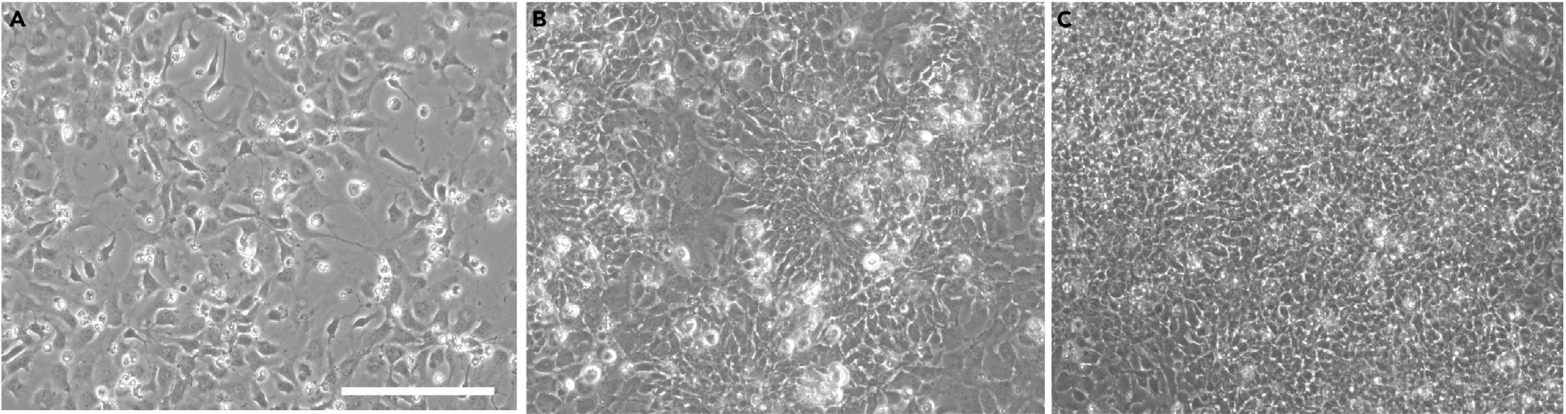
Representative brightfield images at day 14, day 17, and day 20, showing how the culture organizes in the maturation stage of the protocol (A) Image taken at day 14, the first day after plating cells for Stage III. (B) The cells have, by day 17 acquired the morphology of expanded NSCs/NPCs. (C) By day 20 the culture forms a tightly packed and dense small-cell-body population highly reminiscent of a network of NSCs/NPCs and neurons. Images were routinely taken before media changes. As described, daily culture medium replacement is not accompanied with washing steps, thus by day 20, dead cells and debris may be accumulating. Images were taken with an EVOS FL microscope at 20× magnification and scale bar corresponds to 100 μm.

A set of representative phase contrast images at days 14, 17 and 20 showing how the cells organize in maturation stage are shown in Figure 3 (while a set of representative images of the timeline from Day 13–20 is available in Figure 6).

### Immunofluorescence analysis

**Timing: 1 day**

The immunofluorescence analysis was performed using the dilutions described in the key resources table and all the images shown were obtained using the EVOS FL microscope. In brief,

43. Wash cells grown on 13 mm glass coverslips once with 1× PBS and fix in 4% paraformaldehyde for 15 min at room temperature.
44. Wash coverslips 3 times with 1× PBS (10 min per wash).
45. Permeabilize the cells with 0.3% Triton X-100 in 1× PBS for 15 min, wash 3 times with 1× PBS (1 min per wash).
46. Block the cells with 10% horse serum for 30 min.
47. Use anti-FOXG1 primary antibody at a concentration of 1:1000 in 1× PBS containing 0.03% Triton X-100. Dilute all conjugated antibodies 1/500 in 1× PBS containing 0.03% Triton X-100. Incubate coverslips overnight at 4°C (12–16 h).
48. Next day, equilibrate cells on coverslips at room temperature for 2 h and wash 3 times (15-min washes) with 1× PBS, at room temperature.
49. If the coverslips were incubated with a primary antibody, they are then incubated with the secondary antibody in 1× PBS containing 0.03% Triton X-100 at room temperature for 1 h. Then, coverslips are washed 3 times (15-min washes) with 1× PBS, at room temperature.
50. Mount coverslips on microscope slides using the ProLong™ Gold Antifade Mountant containing DAPI to counterstain the nuclei, and according to the manufacturer’s instructions.

### qRT-PCR analysis

**Timing: 10 h**

Perform RNA extraction according to the manufacturer’s instructions; see key resources table for the Qiagen and Nordic Biosite RNA extraction kits and oligonucleotides. In brief,

51. Perform reverse transcription (RT) of total RNA using QuantiTect Reverse Transcription Kit (Qiagen) according to manufacturer's instructions.
52. Amplify cDNA using TaqMan® Gene Expression Master Mix (ThermoFisher Scientific) and Roche or TaqMan gene expression assays for the chosen marker genes. Use oligos for *RPL30* and *RAF1* as normalization controls.
53. The cycling conditions are described below (Table 3).

**Table 3:** cDNA preparation PCR steps

PCR cycling conditions			
Steps	Temperature	Time	Cycles
UNG incubation	50°C	2 min	1
Polymerase activation	95°C	10 min	1
Denaturation	95°C	15 s	40 cycles
Annealing/extension	60°C	1 min	
Hold	4°C	forever
54. Quantification and statistical analysis.
  a. Analyze the resulting Ct-values using the ddCt package v.1.40.0 and visualize using the tidyverse package in R 4.0.4. (Zhang et al., 2021).
  b. Perform statistical comparisons in R using t-test in ggpubr package v.0.4.0 (Kassambara, 2020).

## Expected outcomes

Here we describe the protocol parameters of neuronal differentiation from hESCs towards a heterogenous population of telencephalic progenitors, immature and mature neurons for drug-screening and early brain differentiation studies. For complete details on the analysis of the cells derived and numbers, please refer to Samara et al. (2022). The readers can explore single-cell RNA-seq and ATAC-seq data for four time-points for their genes of interest using the interactive web-tools at https://cancell.medisin.uio.no/scrna/hescneurodiff/ and https://cancell.medisin.uio.no/scatac/hescneurodiff.archr/.

**Figure 4:**
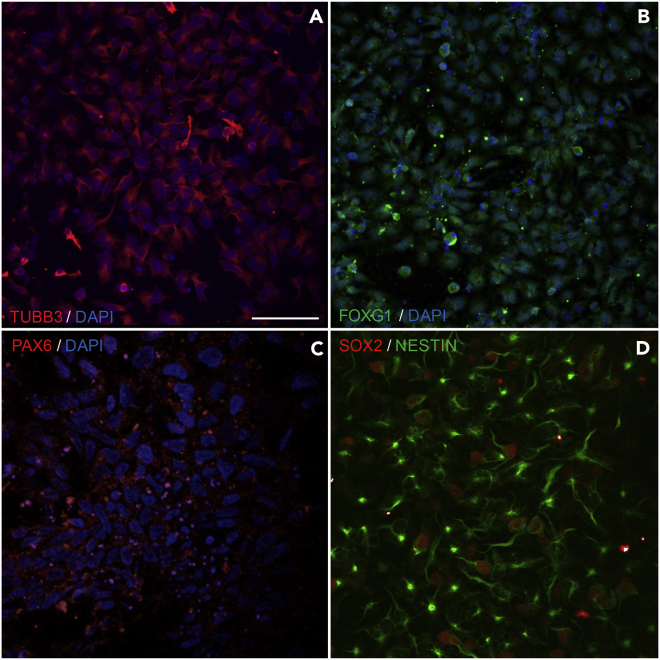
Immunocytochemistry (ICC) / Immunofluorescence analysis at Day 13 To assess the self-patterning stage beyond the characterization by brightfield microscopy, immunofluorescence imaging analysis was performed at day 13. (A) Cells at this stage show abundant expression of the structural cytoskeletal protein expressed in early neurons, beta-3 tubulin (TUBB3 in red) commonly used as an immature neuronal marker (A; DAPI in blue). (B and C) Additionally, cells are immunostained for the early telencephalic marker FOXG1 (B in green; DAPI in blue), and PAX6 (C in red; DAPI in blue). (D) SOX2 is also highly expressed at this stage (D in red) and the progenitor cells also express the intermediate filament NESTIN (D in green). Scale bar corresponds to 20 μm.

**Figure 5:**
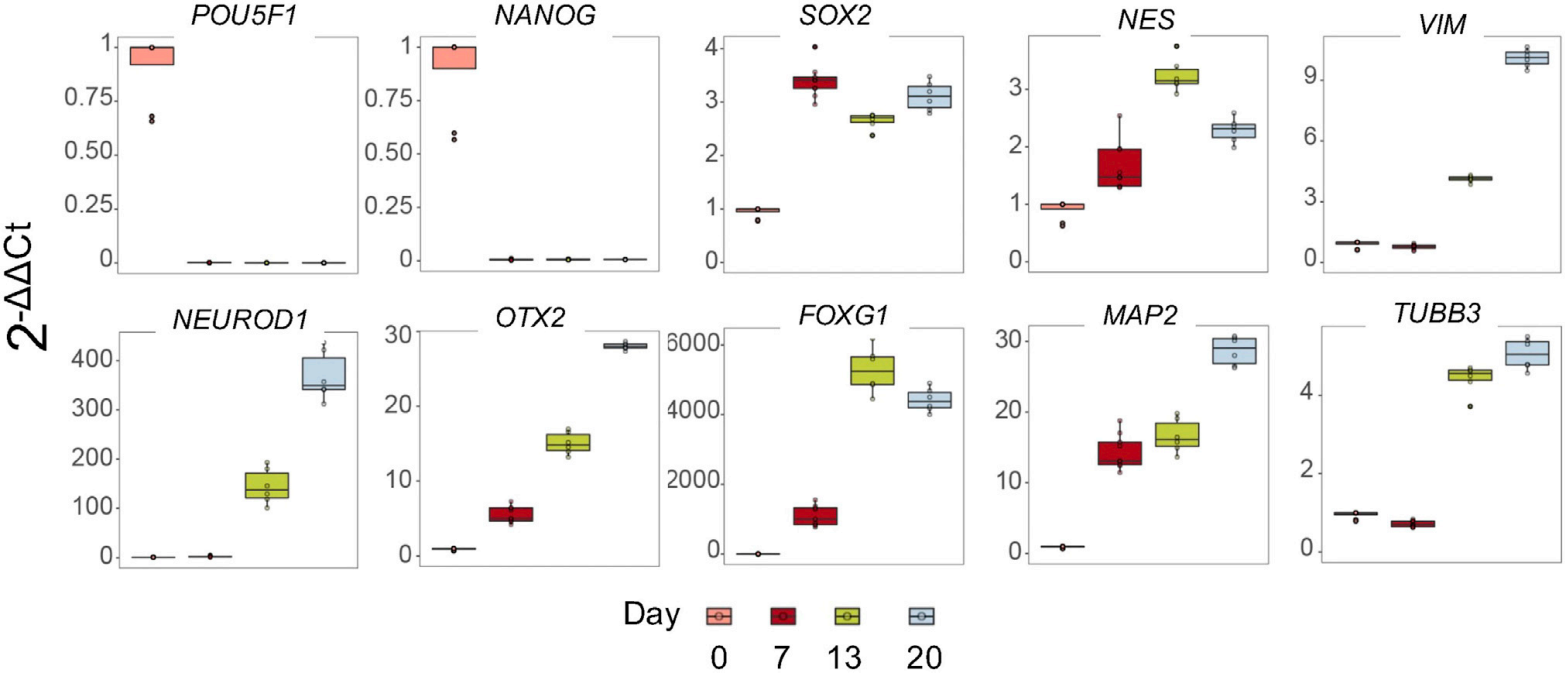
qPCR analysis of pluripotency and neuronal markers In order to characterize the derivative cell populations, hESCs collected at the onset of the protocol (Day 0), and cells derived at all 3 time points of the differentiation protocol (i.e., at Day 7, Day 13 and Day 20) were analyzed for specific pluripotency markers *(POU5F1*, *NANOG*, *SOX2*, *NES*), major neuronal development transcription factors (*SOX2*, *OTX2*, *FOXG1*, *NEUROD1*) and genes related to cytoskeletal rearrangement during differentiation towards neuronal maturation (*NES*, *VIM*, *TUBB3* and *MAP2*) by qRT-PCR. The expression of the pluripotency transcription factors *POU5F1* and *NANOG* decreases to zero already at Day 7, while *SOX2* and *NES* expression increases as hESCs commit to neuronal fate. *NES* expression decreases at Day 20, consistent with an arising neuronal, non-proliferative cell population of Day 20. Similar expression pattern was observed with *FOXG1*, one of the earliest telencephalic specific transcription factors. The expression of the transcription factor *OTX2*, which is known as a regulator of neurogenesis, increases as cells differentiate, promoting their commitment. This increasing pattern of expression from Day 0 to Day 20 is also seen in the expression of the key bHLH enhancer of transcriptional regulators of neurogenesis *NEUROD1*, the mature dendritic marker *MAP2*, the neuronal specific *TUBB3*, and the intermediate filament *VIM*. For the qPCR analysis we used samples collected from 3 separate experiments (cells collected from one well were considered one sample, 3 samples were collected per experiment, and each sample collected for analysis was run in technical triplicate.) Each value is plotted as a point (average value of technical triplicates), on top of the box plot. The box represents 25th to the 75th percentile, and the midline represents the median value, and the line goes from minimum to maximum.

**Figure 6:**
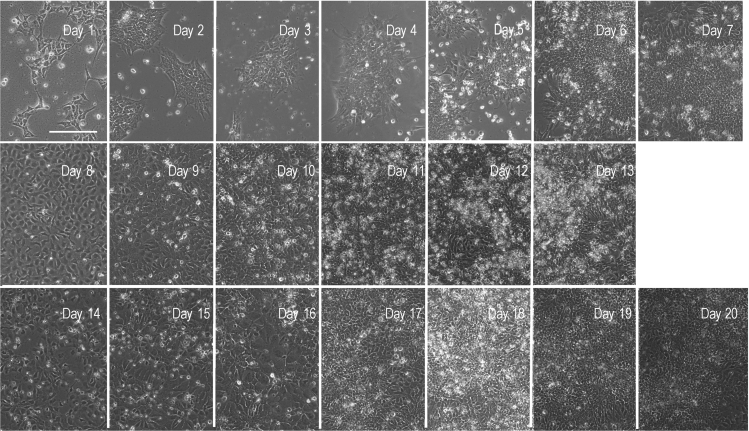
A timeline of differentiation for HS360 hESCs Representative phase contrast images of cells through differentiation (Days 1–20). The images were taken with the EVOS FL microscope, and the scale bar corresponds to 100 μm.

**Figure 7:**
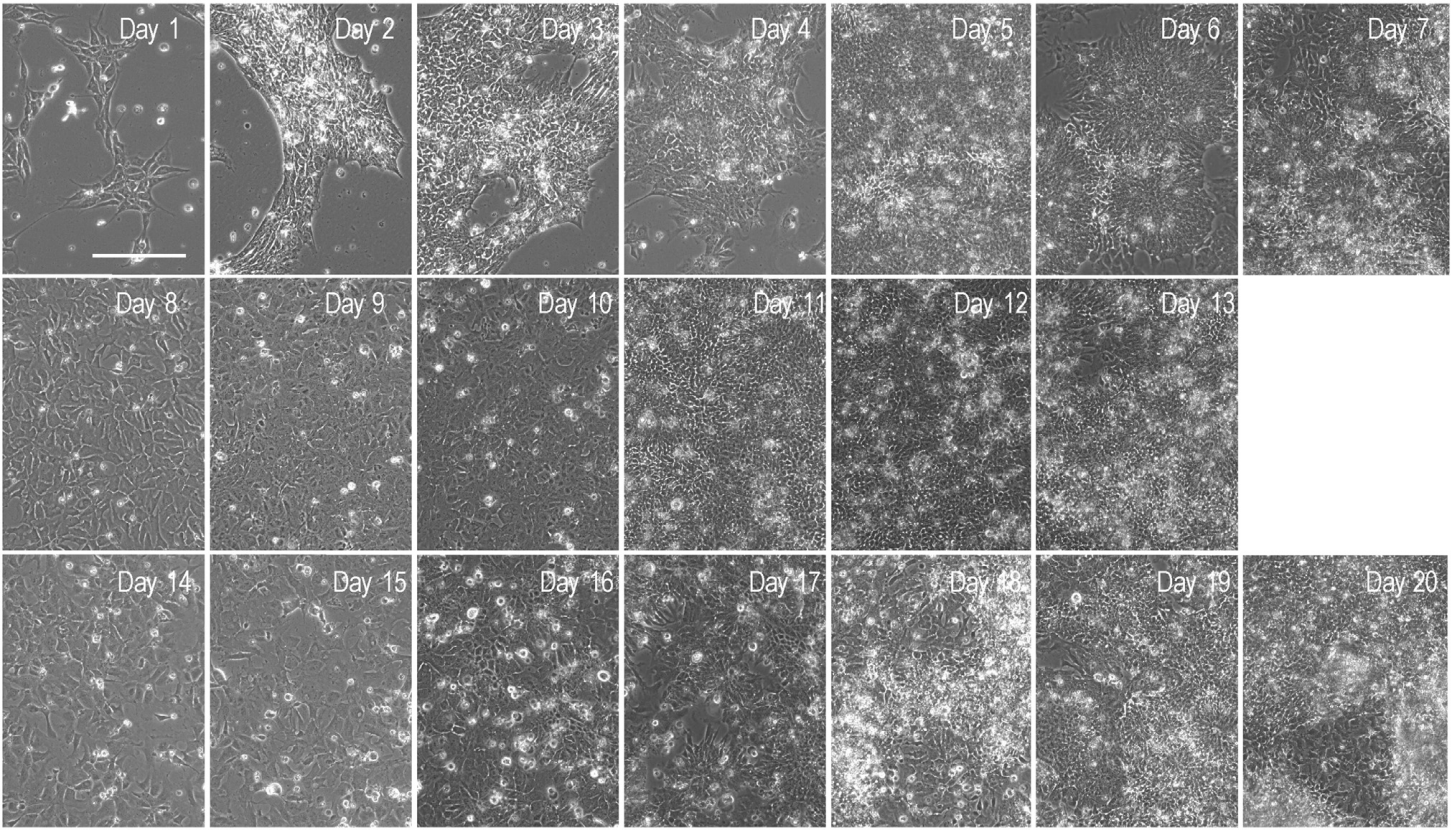
A timeline of differentiation when the protocol was replicated using H9 hESCs Representative phase contrast images of cells through differentiation (Days 1–20). The images were taken with the EVOS FL microscope, and the scale bar corresponds to 100 μm.

Visual aids, such as phase contrast and ICC images of the differentiating cells, and gene expression analyses are made available for this protocol. For reproducibility purposes we share representative immunofluorescence images of the cell cultures after the self-patterning stage (Stage II; Figure 4). Furthermore, indicative information on the total RNA and genomic DNA yield per time point is presented at Table 4. In addition, qRT-PCR analysis of pluripotency markers, major neuronal development transcription factors and genes related to cytoskeletal rearrangement during differentiation towards neuronal maturation are shown in Figure 5. Finally, representative images of the differentiating cells at all days and stages are presented in Figure 6, whereas Figure 7 shows the timeline of differentiation when the protocol was replicated using H9 hESCs.

**Table 4:** Indicative RNA and DNA total yields after the respective nucleic acid isolation and extraction at days 0, 7, 13, and 20

Day	Genomic DNA yield (μg)	Total RNA yield (μg)
0 (6-well plate)	1–4	21–38
7 (12-well plate)	2–5	10–17
13 (12-well plate)	2–5	7–17
20 (12-well plate)	2–8	11–24

### Advantages of the protocol

Neural induction and differentiation in a monolayer offer the advantage of more homogeneous cell differentiation without the aggregation steps of 3D cultures. Undoubtedly, 3D culture models are the advanced option to study brain architecture and recapitulate the complexity of intraneuronal connectivity. However, this protocol was designed as a neurotoxicology platform aiming to facilitate studies of early brain development events. The protocol has been reproduced with HS360 and the more commonly used H9 hESCs. Some optimization might be required for other hESC and iPSC cell lines.

This protocol has been optimized for use with widely available coating agents and matrices, previously tested neuronal induction reagents, and standardized cell numbers. In addition, cell numbers and substrate-coating conditions have been optimized to minimize cell passaging and thus to avert mechanical disruption of the cell connections in the monolayer after mechanical cell collection. The daily media-changes are suggested to reduce the possible bias conferred on neurotoxicology experiments by compound instability and degradation in cell culture conditions, which could mask the effect of exposure to the compounds of interest.

### Drug treatment of differentiating cells

This neuronal differentiation protocol is optimized for drug/toxicology treatments and concentration screening with daily media changes as some compounds have short half-lives. hESCs are sensitive to high drug concentrations, and neural induction is a phenomenon that may induce apoptosis. High drug concentrations may thus result in excess cell death manifesting with floating cell debris due to cell death at various stages of the protocol. It may cause a major lag in the neural induction stage of the protocol (Stage I), and the cells might not follow the self-patterning stage (Stage II).

## Limitations

Although it is expected that a proportion of the cells at Day 20 corresponds to mature neurons, the membrane electrochemical maturation properties, secretion of neurotransmitters of the neurons generated by this protocol have not been assessed. Thus, we cannot at this stage provide more relevant information regarding these properties. However, the scRNA-seq data that correspond to the cells generated are available at https://cancell.medisin.uio.no/scrna/hescneurodiff/.

## Troubleshooting

### Problem 1

Some users are less experienced with human embryonic stem cells (hESCs) or neuronal differentiation studies.

### Potential solution

After thawing hESC stock vials, hESCs are routinely kept in culture for 2-3 passages, and a 6-well cell culture plate is regularly used for hESC maintenance. In these conditions, using E8 as the cell culture medium, at 75-90% confluency, over a million HS360 cells can be seeded from a well of a 6-well plate. Reproducibility of this protocol depends on accurate cell counts and high cell viability (steps 2-7, 12-16, 25-29 and 38-42).

Moreover, Rock inhibitor is added after thawing or passaging hESCs and NPCS/ NSCs, as it has been shown to improve both the recovery of cryopreserved and growth upon subculture of hESCs (Claassen et al., 2009).

hESCs in this protocol are maintained in antibiotic-free conditions. As hESCs are very sensitive to CO_2_ and temperature changes, they may detach from the culture vessel if these fluctuate (step 1-7). Thus, CO_2_ and temperature should be checked regularly and, if possible, a dedicated cell incubator should be used.

The times for cell detachment using Accutase refer to Accutase prewarmed to 37°C. hESCs in this protocol are maintained in antibiotic free conditions. For all centrifugation steps, the temperature was set to room temperature and the conditions (for cell collection and washing) were 300 x *g* for 4 minutes.

### Problem 2

The protocol is standardized for a 12-well culture plate format.

### Potential solution

The protocol was optimized for a 12-well culture plate format, allowing for smaller volumes of media, but still providing enough cells for RNA or protein isolation. In a 12-well format, 4 biological triplicates can be tested per plate (for example a triplicate of wells treated under control conditions and 3 triplicates of 3 different doses of the drug treatment of choice). 24-well plate format volumes and cell seeding counts are also described to downscale costs or to use in pilot experiments. Additionally, 13 mm glass coverslips can be placed in the 24-well dishes facilitating immunofluorescence or other type of or microscopy experiments. Upscaling to 6-well plates for differentt PSCs might require some optimization, beyond the obvious surface area conversion.

### Problem 3

After cell collection and replating, there is uneven cell spreading in the dish (steps 4, 6, 18, 30 and 41).

### Potential solution

Slide the plate back and forth, left and right about 5 times in each direction, avoiding circular motions that could cause cells to roll back into the centre of wells. Instead, dishes should be moved horizontally (5 times), and side-to-side (5 times), slowly, and carefully moved to the cell incubator.

Plasticware from different companies has been tested with no effect on cell cultures under the recommended coating conditions.

## Resource availability

### Lead contact

Further information and requests for resources and reagents should be directed to and will be fulfilled by the lead contact ragnhild.eskeland@medisin.uio.no.

### Materials availability

This study did not generate new reagents.

## Data Availability

Data reported in this paper will be shared by the lead contact upon request. This study does not report original code.
